# Science and Pseudo-Science: The Hereditary Criminal Again?

**Published:** 1917-05-12

**Authors:** 


### SCIENCE AND PSEUDO-SCIENCE.

### The Hereditary Criminal Again?

Another most valuable contribution to the *Journal of Mental Science* is made by Sir Bryan Donkin in a paper entitled "Notes on Mental Defect in Criminals." Since Lombroso first started the systematic study of criminals, the most extraordinary and pernicious notions have been prevalent with respect to them. It is difficult to say which is the more to be wondered at—the absurdity of the views that have been taught, and that still are taught, or the avidity with which they have been received and adopted, and their consequences put into practice. The underlying reason for this gullibility of the public, and of many intelligent men who ought to know better, and who do know better if they would only exercise their wits, is that these absurd views are called " scientific." In these days " scientific " is as much a word to conjure with as " abracadabra " was in days that have passed away. The great majority of people in this country, and the enormous majority of what are called the educated classes, are as ignorant of science as they are of Sanskrit—some of them a good deal more ignorant. They do not even know that what is meant by " scientific " is not necessarily " true " or " certain," but merely means arranged in a methodical and systematic manner. Many things that are called scientific are, when tried by this test, not scientific at all; and it is just as easy to arrange error in a methodical and systematic manner as to arrange truth. A very great deal of science is erroneous, as is proved by the fact that it is constantly being corrected, and a doctrine is no more to be accepted because it is called scientific than because it is called orthodox. The one is as often wrong as the other. There are certain principles and methods of investigation that are pursued by the most careful and conscientious and intelligent men who work at science, and these principles and methods are usually called scientific, and may properly be so called; but in the first place science has no monopoly of them, for careful and intelligent and conscientious men devised them, and used them in their business transactions long before they were utilised in science, and in the second place many men who investigate the recondite phenomena that are called scientific do so in a manner that is anything but scientific. It was thus with Lombroso. He investigated the physical and mental and moral qualities of criminals with great industry, but he did not investigate them in a scientific manner, for, in the first place, he very soon formed an hypothesis which he determined to establish by hook or by crook, and having once adopted it he looked for only such evidence as corroborated and supported it, and disregarded all evidence that conflicted with it. This method is unfortunately employed by many men who work at subjects usually called scientific, and are therefore called scientific men, which they are not. Lombroso taught that criminals are racial degenerates, that they are in nature different from the non-criminal community, and that the difference in nature consists in a reversion to the savage, or barbarous, or childish state; so that a criminal is from birth different from people who are not criminals. He is a peculiar being and owes his peculiarity to an accident of birth as much as does the albino, or the red-haired, or the dolichocephalic person. His criminality is born in him, it is hereditary, innate, constitutional, and as on the one hand it cannot be eliminated, so on the other he is not to be held responsible for his criminal acts. Any doctrine more pernicious to the well-being of society, and even to the existence of society, it would be difficult to devise. It is so inherently and manifestly wrong that it is now discarded; but though it is formally discarded, it has left behind it certain remnants that cling tenaciously in the minds of those who term themselves criminologists, and still has effects that are disastrous in proportion as this doctrine is put into practice. It is discarded because it is inherently and manifestly wrong in that it is opposed to universal experience. It flatly contradicts the Shakespearean maxim, "How oft the sight of means to do ill deeds makes ill deeds done!" If Lombroso's doctrine were true, this could never happen. No opportunity, no temptation would have any effect upon the man who is not degenerate. No prosperity, no absence of temptation, opportunity, or motive could keep from crime the criminal who is a born criminal. Stated thus, even Lombroso would scarcely subscribe to his own doctrine, but the pseudo-scientific, who have an hypothesis to establish at any cost, do not trouble to trace out the consequences of their doctrine. Sir Bryan Donkin's paper is so full of interesting matter that we shall be tempted to return to it again.

